# Supplementary material for: Evidence supported by Mendelian randomization: impact on inflammatory factors in knee osteoarthritis
Source: Front Med (Lausanne). 2024 May 28;11:1382836. doi: 10.3389/fmed.2024.1382836 (PMC11165061; doi:10.3389/fmed.2024.1382836)
Supplement: Supplementary file 1 [file Data_Sheet_1.ZIP › data/GCST90274789_5e6/789.leaveoneout.pdf]

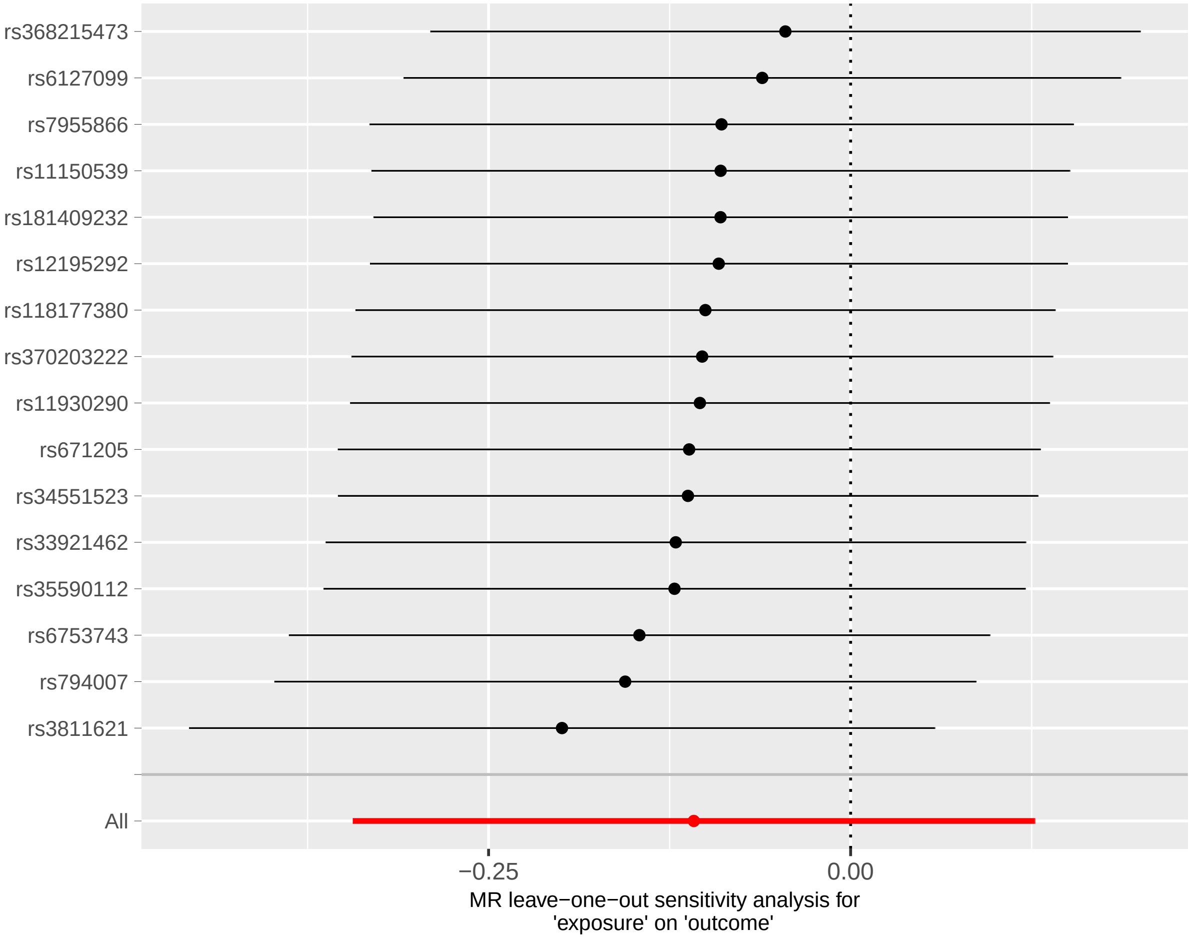
rs368215473
rs6127099
rs7955866
rs11150539
rs181409232
rs12195292
rs118177380
rs370203222
rs11930290
rs671205
rs34551523
rs33921462
rs35590112
rs6753743
rs794007
rs3811621
All
−0.25
0.00
MR leave−one−out sensitivity analysis for
'exposure' on 'outcome'
